# Supplementary material for: Control of membrane barrier during bacterial type-III protein secretion
Source: Nat Commun. 2021 Jun 28;12:3999. doi: 10.1038/s41467-021-24226-1 (PMC8239009; doi:10.1038/s41467-021-24226-1)
Supplement: Supplementary file 1 — Supplementary Information [file 41467_2021_24226_MOESM1_ESM.pdf]

Supplementary Information

Control of membrane barrier during bacterial type-III protein secretion

Svenja Hüsing[1,2], Manuel Halte[1], Ulf van Look[1,2], Alina Guse[1,3], Eric J. C. Gálvez[2], Emmanuelle Charpentier[2], David F. Blair[4], Marc Erhardt[1,*], and Thibaud T. Renault[1,2,5,6,*]

[1]Institute for Biology – Bacterial Physiology, Humboldt-Universität zu Berlin, Philippstr. 13, 10115 Berlin, Germany
[2]Max Planck Unit for the Science of Pathogens, Charitéplatz 1, 10117 Berlin, Germany
[3]current address: Department of Molecular and Cellular Biology, Harvard University, Cambridge, MA 02138, USA
[4]School of Biology, University of Utah, Salt Lake City, UT 84112, USA
[5]CNRS, UMR5234, Université de Bordeaux, 146 rue Léo Saignat, 33076 Bordeaux, France
[6]Institut Européen de Chimie et Biologie, Université de Bordeaux, 2 rue Robert Escarpit, 33607 Pessac, France
[*]email: thibaud.renault@cnrs.fr and marc.erhardt@hu-berlin.de

doi:10.1038/s41467-021-24226-1

Supplementary figures

Figure S1 1
Figure S2 2
Figure S3 3
Figure S4 4
Figure S5 5
Figure S6 5

Supplementary tables

Table S1 list of suppressors (charged M-loop) . . 6
Table S2 list of suppressors (XXX M-loop) 6
Table S3 list of strains and plasmids 7
Table S4 list of primers 9

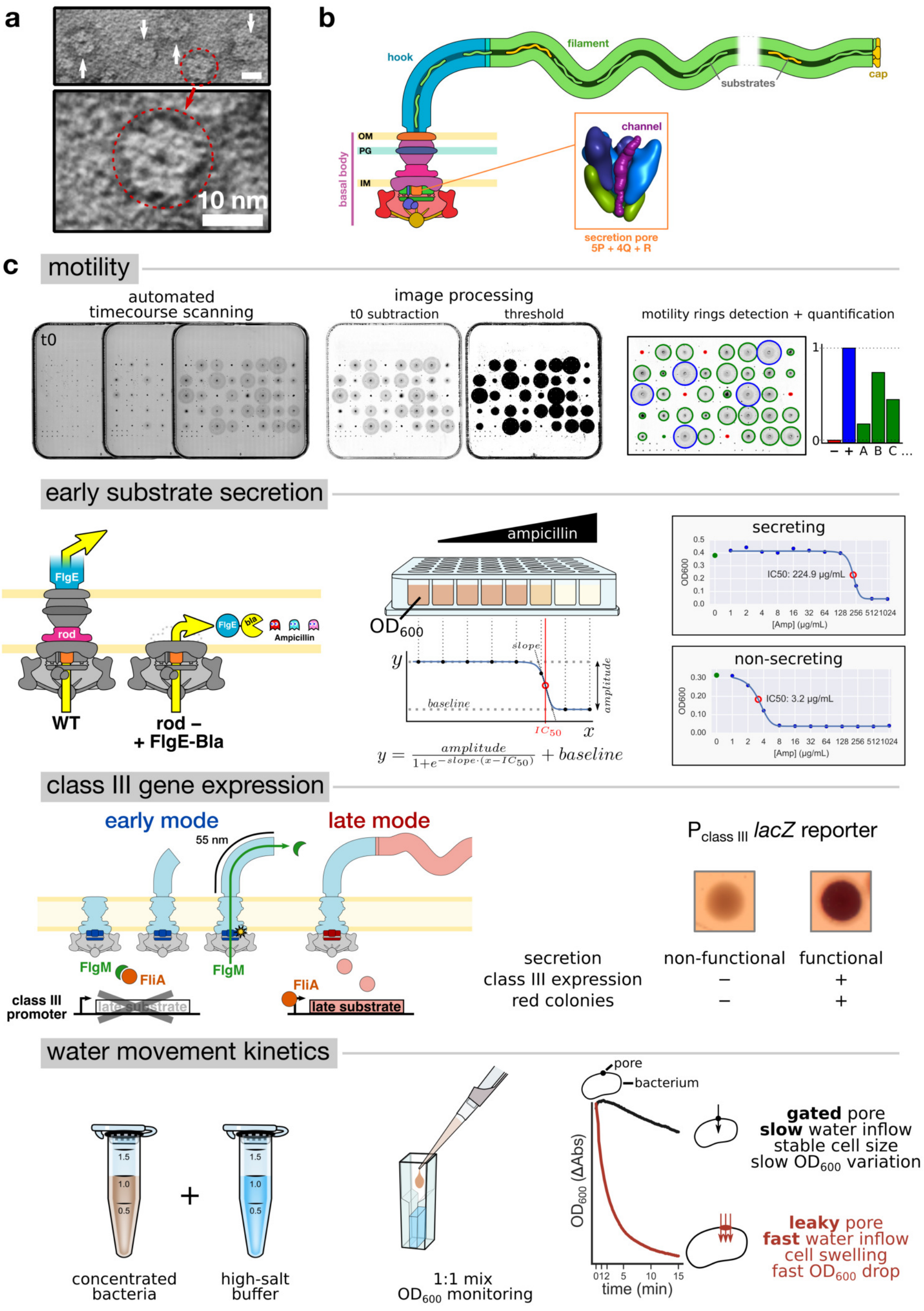

Figure S1. **a.** Over-expressed FliP form oligomers reminiscent of a pore. **b.** Location of the T3SS secretion pore in the assembled flagellum. The FliP1, FliQ1, and FliR subunits are hidden to better visualize the inside of the pore. The channel (shown in purple) was computed using pymol and Caver 3 (see Figure 2 in the main text). **c.** Schematic description of the assays used in this study. **Motility:** Bacteria are inoculated in soft agar and plates are monitored for the apparition of motility rings. Scanned images of the motility plates are processed to subtract background and detect the contour of the motility rings. Quantification is done by measuring the average radius of the motility rings and using ΔfliP and WT FliP strains as negative and positive controls, respectively. **Early substrate secretion:** Deletion of the rod proteins FlgB/C enables secretion of the flagellar substrates in the periplasmic space. Fusion of the β-lactamase TEM-1 (devoid of its secretion signal) to an early flagellar substrate (the hook protein FlgE), renders the cells resistant to ampicillin to a level that is proportional to the secretion of the fusion substrate. The level of ampicillin resistance is quantified by monitoring bacterial growth in presence of increasing concentrations of ampicillin, and by fitting the observed data to a logistic function. The fitted IC50 parameter represents the antibiotic concentration that inhibits bacterial growth by 50%. **Class III gene expression reporter:** Flagellar assembly occurs in two consecutive steps. First the basal body and hook (early substrates) are assembled, and during this time expression of the late substrates is repressed by the anti-sigma factor FlgM. Upon completion of the hook, FlgM is secreted and relieves the inhibition of flagellar class III promoters, that control the expression of late substrate, to enable assembly of the flagellar filament. Placing the *lacZ* gene under the control of a class III flagellar promoter allows to report the successful assembly of the hook-basal body, and thus the proper function of the T3SS secretion pore. Functional clones appear red on MacConkey plates. **Water movement kinetics (secretion pore leakage):** Bacteria expressing different variants of the T3SS pore are washed and concentrated in 1×PBS. The bacteria are then diluted 1:1 in a high salt buffer. Variants bearing a functional, sealed, T3SS pore are resistant to passage of water across the pore and their cell size thus remains stable. Conversely, variants with a defect of pore gating enable a flow of water across the pore, which results in cell swelling and a rapid drop of optical density. The underlying principle of this assay is detailed in (1).

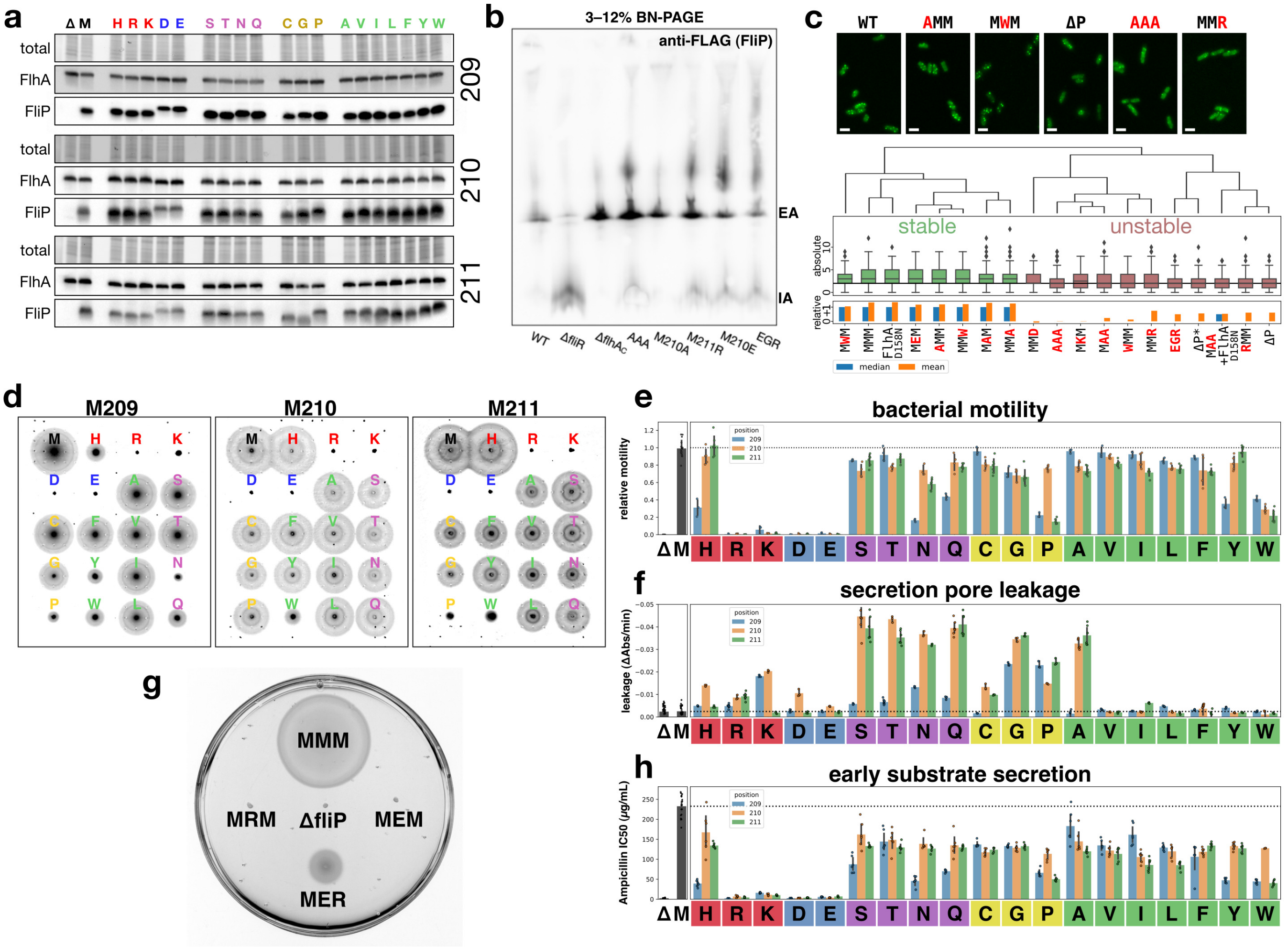

Figure S2. a. All single amino-acid substitutions in FliP MMM (209–211) are expressed and localized to the membrane. Western-blot of membrane extracts. A 3×FLAG tag and flexible linkers (SAGASA-DYKDHDGDYKDHDIDYKDDDDK-SAGASA) were inserted in a non-conserved region (after G157) to allow detection of the protein. Membrane fractions were prepared by mechanical disruption of the cells and differential centrifugations. TCE staining of total proteins and detection of the membrane flagellar protein FlhA were used as loading controls. Furthermore, FlhA assembly requires the prior correct assembly of FliP (2). n>2 biological replicates. **b.** BN-PAGE of membrane fractions solubilized in DDM. The WT and stable mutants efficiently assemble the export apparatus (EA). The AAA, M211R, M210E, and EGR M-loop mutants are partially unstable and display a mix of EA and of intermediates of assembly (IA). ΔfliR is used as a control as the mutation prevents assembly of the export apparatus. n=2 biological replicates. **c.** Top: fluorescence images of FlhA-mNeonGreen foci for example FliP mutants. The scale bars represent 2 μm. Bottom: automated numbering of the FlhA-mNeonGreen foci as a proxy of EA stability. Both the absolute number of foci and the number relative to the Δ*fliP* (ΔP) control strain (that does not assemble the export apparatus) are represented. The boxplot show the median and the quartiles, the whiskers extend to 1.5 time the inter-quartile range (IQR) around the median, and the points are the outliers outside of the 1.5 IQR. The tree was calculated from the matrix of two-sided pairwise T-tests between conditions (p-values were corrected using the Benjamini/Hochberg method to account for multiple testing). The strains cluster into two major groups which we categorized as stable and unstable. All strains carry a Δ*flgBC* mutation, except for Δ*P**. **d.** Motility in soft agar of all single variants of the M-loop. Scanning of the plates was automated and background was subtracted from the initial time point. **e—f.** Aggregated data of the quantification of several replicates of the motility (**e**) and leakage (**f**) assays as described in Figure S1c. Assembly of the flagellum and maintenance of membrane barrier are two crucial functions of the T3SS secretion pore that are affected by the presence of certain residues in the M-loop. In e: n=3 biological replicates for the M209 mutants, except for M209T (n=4); n=5 biological replicates for the M210 mutants, except mutations to T/S/Q/F (n=4) and N (n=3); n=7 biological replicates for the M211 mutants, except for M211S (n=6) and M211T (n=4). In f: n=3 biological replicates for all mutants, except M209R/T (n=9), M210S/F (n=8), M210Q (n=7), M209A-M210A-M211A/R/S/T/Q/F (n=6); WT (n=30), Δ*fliP* (n=33). **g.** Motility in soft agar of two non-motile charged variants of the M-loop (M210E and M210R), and of a MER revertant that derives from the M210E background. The WT (MMM) and a strain lacking FliP are shown as positive and negative controls, respectively. **h.** Secretion in the periplasm of a fusion between the hook protein (FlgE) and the β-lactamase TEM-1 devoid of its signal peptide renders the strain resistant to ampicillin.

Δ: Δ*fliP* mutant that cannot assemble the fT3SS; M: WT strain with a MMM M-loop motif.
In panels e, f, h, the bars represent the mean value of the individual measurements, the error bars the standard deviation, and the dotted lines the physiological (WT) values.
Amino acids are colored according to classical physicochemical groups (red: positively charged, blue: negatively charged, purple: polar, yellow: special, green: hydrophobic).

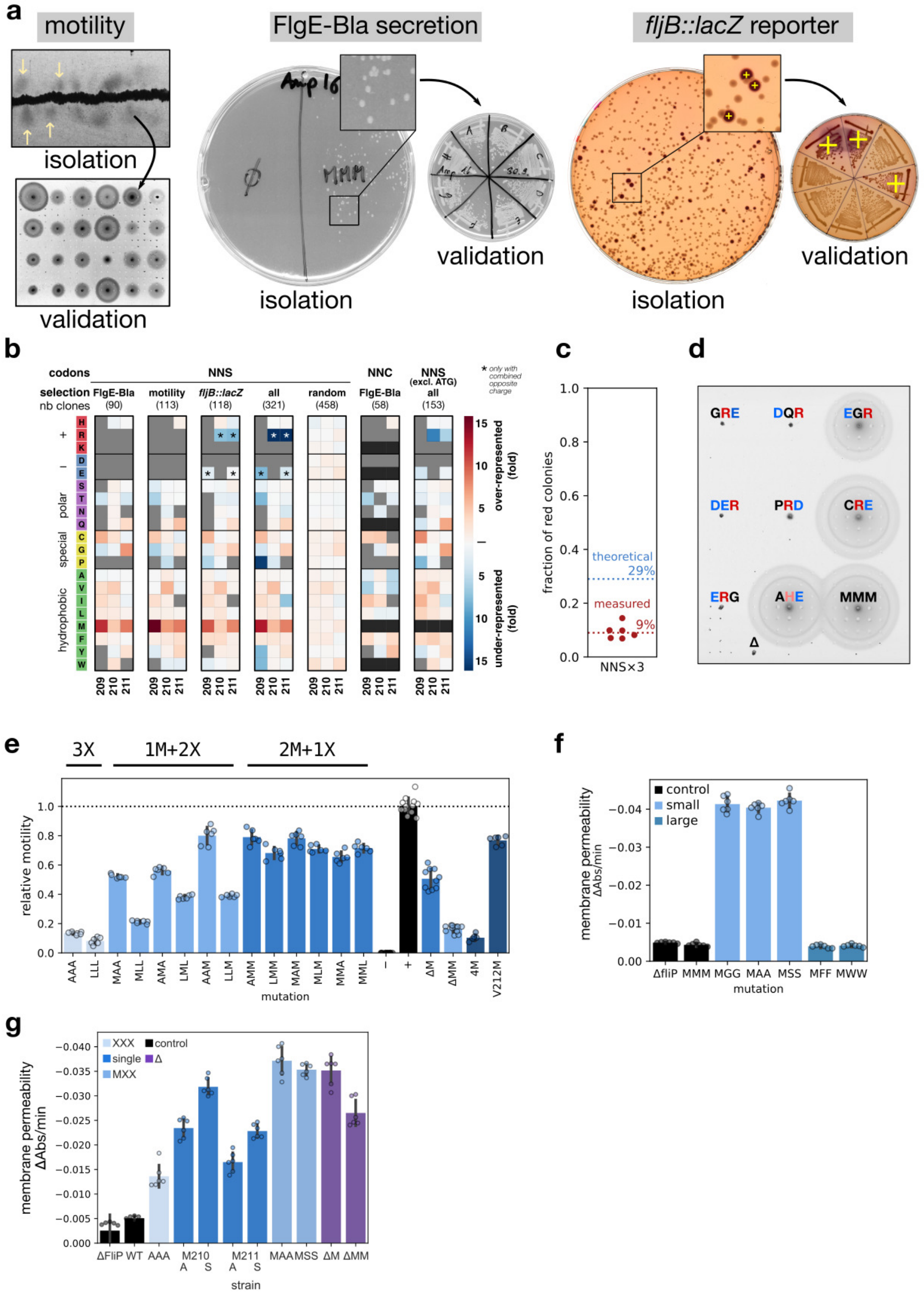

Figure S3. a. Independent methods of functional random M-loop variants selection. All methods are performed after recombination of a degenerate NNS×3 oligonucleotide in place of the M-loop. **Left:** motility selection relies on the successful assembly of the bacterial flagellum, which confers the ability to swim away from the inoculation site. **Middle:** FlgE-bla secretion confers ampicillin resistance only when the T3SS is able to secrete the fusion substrate. **Right:** In this reporter system, clones are first selected for successful recombination. Functional clones are identified using a Mu*d*A transcriptional fusion to the flagellin *fljB*, that triggers β-lactamase expression upon completion of the flagellar hook-basal body. Positive clones appear red on MacConkey agar. **b.** Frequency of amino-acid use per position in the random M-loop variants. From left to right: FlgE-bla/motility/*fljB::lacZ*: three independent methods of selection for functional motifs. All: combination of the result of the three selection methods. Random: absence of selection for fT3SS function. Those last two datasets indicate that in absence of methionine a slight over-representation in medium size hydrophobic amino acids (all positions), cysteine at position 209, and glutamine/glycine (position 211) is observed, without reaching the level of methionine. NNC/FlgE-bla: FlgE-bla based selection using an NNC×3 degenerate oligonucleotide to avoid methionines. NNS excl. ATG: exclusion of the motifs that do not contain methionine. **c.** The *fljB::lacZ* reporter system enables to measure the proportion of functional M-loop variants by counting the proportion of red colonies. Using an NNS×3 recombination oligonucleotide and excluding amino-acids not tolerated at each position (209: HRKDEWYPNQ (13 codons), 210: RKDEW (7 codons), 211: RKDEWP (9 codons), see. Figure 2B) plus one stop codon, a functional motif would be obtained with a probability 18/32×24/32×22/32 = 0.29, which is much higher than the observed frequency. This suggests that not only the nature of the residues at each position, but also their combination has an effect on the function of the M-loop. **d.** Example of M-loop motifs with a combination of negative and positive charge. Depending on the position and residues, the phenotype can be non-motile (GRE, DQR, DER, PRD, ERG) or motile (EGR, CRE, AHE), which confirms that a net charge in the M-loop does not allow T3SS function. **e.** Combination of methionines and leucines or alanine indicate that T3SS function is roughly proportional to the number of methionine. ΔM/ΔMM: deletion of 1 or 2 methionines in the M-loop. 4M: insertion of an extra methionine in the loop. V212M: substitution of the valine 212 that is immediately after the M-loop to mimic the MMMM motif from the SpaP protein in the vT3SS-1 of *S.* typhimurium. n=6 biological replicates except for -/ΔP and +/WT (n=12) and for ΔM (n=10). **f.** Among the MXX M-loop mutants, only variants to residues with small side chain induce a leakage through the secretion pore. n=6 biological replicates. **g.** Single mutations to serines induce a stronger leakage than their alanine counterparts, indicating that leakage is favored by hydrophilicity and hindered by hydrophobicity, as expected. Unlike triple mutations, double mutations of only the two exposed methionines reached a maximum of leakage similar to that observed in our previously reported single methionine deletion mutant (ΔM). A double deletion mutant (ΔMM) was however less leaky, likely resulting from a structural effect rendering the complex unstable as observed for the AAA mutation. n=6 biological replicates.
In panels e, f, g the bars represent the mean value of the individual measurements and the error bars the standard deviation.

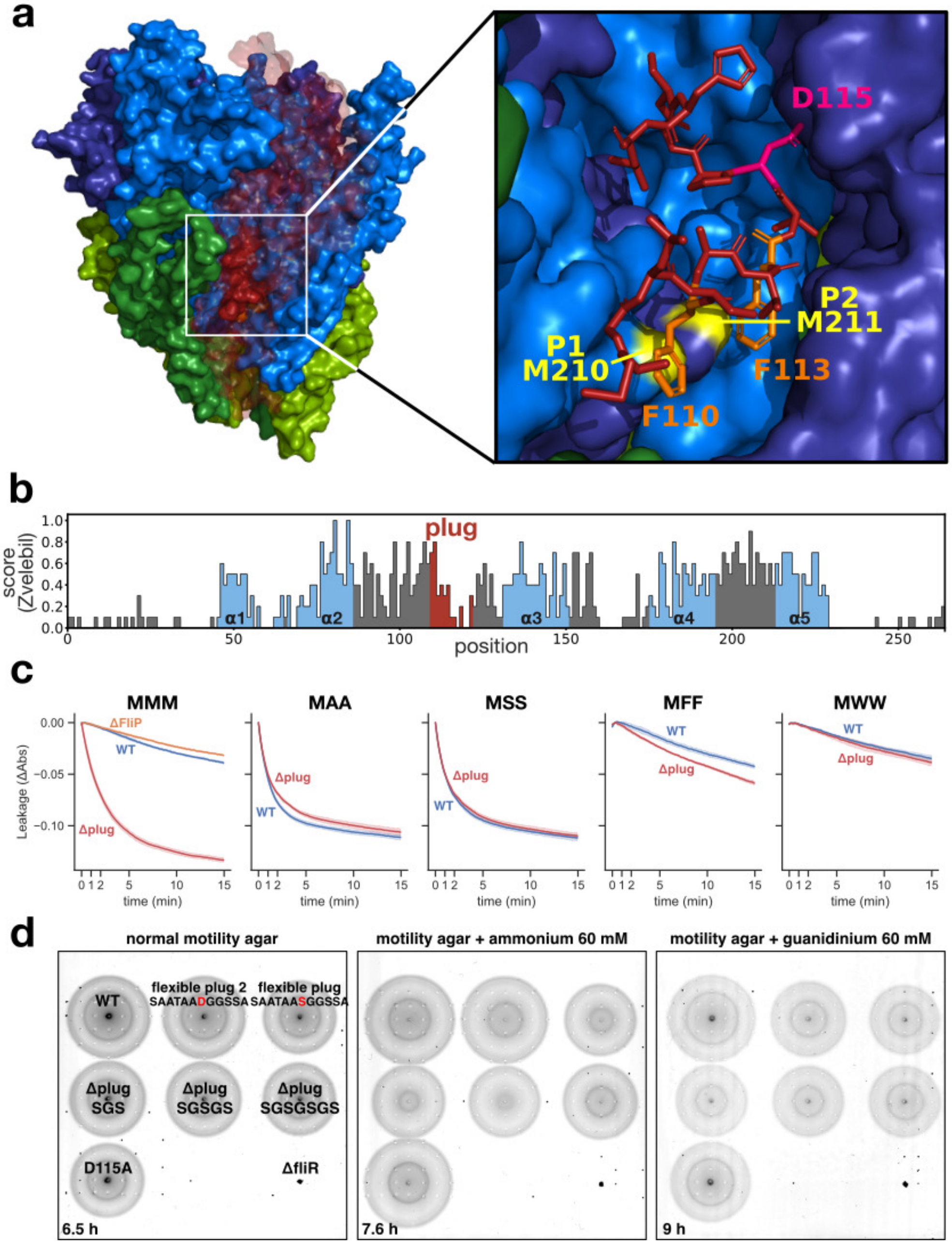

Figure S4. a. The plug domain of FliR is in direct interaction with the M-gasket. In particular, two mildly conserved hydrophobic residues (F110 and F113) are in contact with two methionines (M210 of P1 subunit and M211 of P2 subunit). Another residue of the plug D115, that is highly conserved, is also in interaction with T80 in the P4 subunit. Only the plug residues of FliR are represented for clarity. **b.** The plug domain of FliR displays a low Zvelebil amino-acid conservation score, which reflects a high diversity of amino-acid properties among homologs (3). **c.** Leakage of the fT3SS for combinations of FliP and FliR mutations. The combination of the plug of FliR and the M-gasket is required to maintain membrane barrier. Failure of either of the domains is sufficient to enable leakage of small molecules through the secretion pore. **d.** Motility in soft agar, with or without addition of 60 mM ammonium/guanidinium chloride in the medium, reports that various mutations of the FliR plug do not result in a marked alteration of flagellum assembly and function.

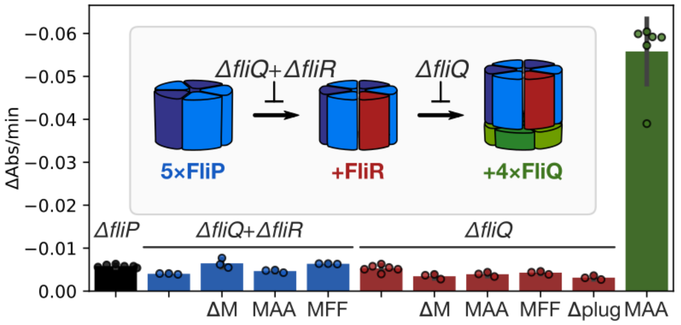

Figure S5. The ΔM (one of the three methionines was removed) and MAA M-loop mutants of FliP, and Δplug mutant of FliR, all of which cause leakage, were used as reporters to assess whether intermediates of assembly of the FliPQR complex require membrane gating. FliPQR assembly was locked into intermediate states by deletion of *fliQ* or *fliQ*+*fliR*. The FliP MFF mutant was used as a non-leaky control. The bars represent the mean value of the individual measurements and the error bars the standard deviation.

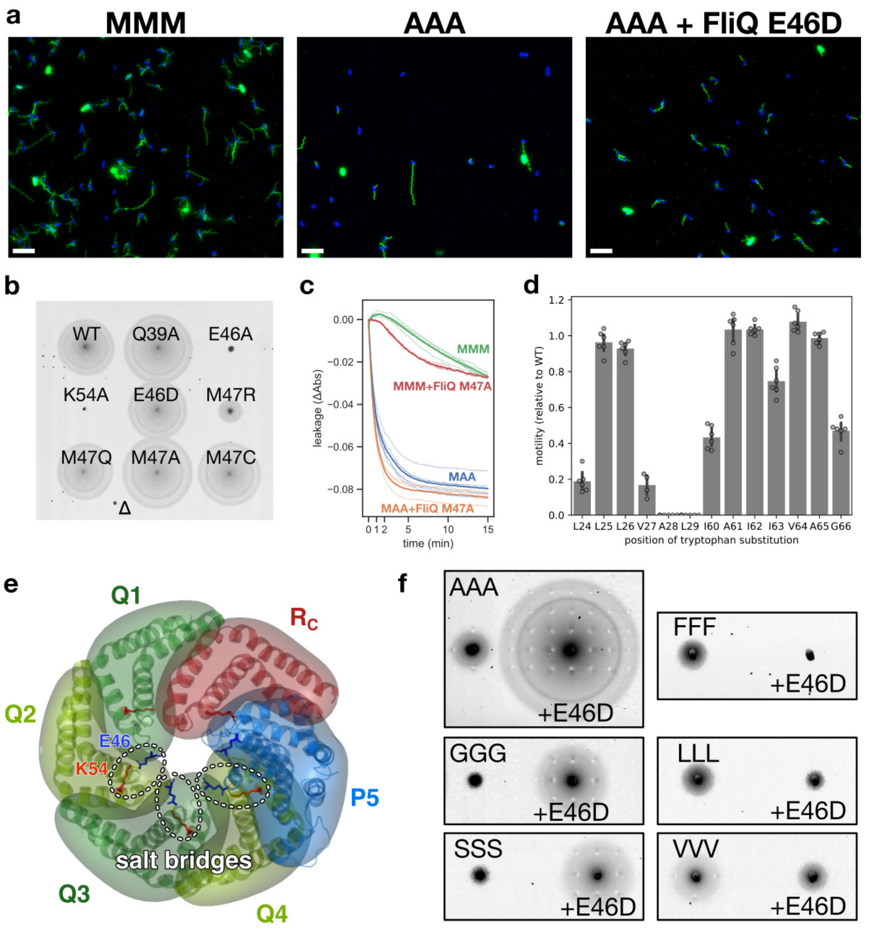

Figure S6. a. Representative images of flagellum immunostaining (green color). The AAA M-loop mutant has a structural defect and can assemble less filaments than the WT (MMM), thus resulting in altered motility. The bacteria are stained with DAPI (blue). The scale bars represent 10 μm. n=5 biological replicates. **b.** Motility in soft agar of various FliQ mutants. The E46-K54 residues involved in an inter-subunit salt bridge are required for T3SS secretion pore function. Conversely, the Q39 and M47 residues do not play a crucial role. **c.** Leakage of the fT3SS for combinations of FliP and FliQ mutations. The M47 of FliQ does not play a critical role in fT3SS secretion pore gating. **d.** Tryptophan scanning of the central parts of FliQ α-helices. The A28W and L29W mutations abrogate motility. n=6 biological replicates, except for V27W (n=4). The bars represent the mean value of the individual measurements and the error bars the standard deviation. **e.** The three FliQ inter-molecular salt bridges are located close to the mouth of the channel and participate in maintaining the stability of the M-gasket. **f.** Motility in soft agar. The FliQ E46D mutation improves motility of the AAA/GGG/SSS M-loop variants, but further impairs that of the FFF/LLL/VVV variants.

Table S1. Suppressors of motility defect of M-loop variants with charged residues. Non motile FliP mutants bearing a charge in the M-loop were inoculated in soft agar and plates were monitored until flares of motile suppressors were observed. The resulting suppressors were isolated and sequenced.

Original mutation	Suppressor mutation	Biochemical effect
M210E	MER	neutralization of the net charge
	ΔMM (×5)	removal of the charged residue
M210R	M210H	
	M210L (×2)	
M210D	ΔMM	
	M210G	

Table S2. List of mutations in known regulators of flagellum assembly that enhance function of impaired FliP M-loop variants. Poorly motile FliP M-loop variants (AAA, GGG, VVV, and SSS) were inoculated in soft agar and suppressors were isolated. Regulators that were identified independently in several backgrounds or experiments are displayed in bold. The HGVS nomenclature is used.

Regulator	Function	Identified mutations and matching M-loop variants	
flhDC promoter	promoter of the flagellar master regulator flhDC	c.-195A>G	AAA
		c.-198A>G	GGG
		c.-196_-195insA	AAA
		c.-211G>A	GGG, VVV, SSS
lrhA	negative transcriptional regulator of flhDC (4)	p.Ala30del	AAA
		p.Ala30_Ala31del	AAA, SSS
		p.Asp12_Leu13del	AAA
		p.Leu11_Asp12insAspLeu	AAA
		p.Ala29dup	AAA
		p.Ala29_Ala30insAlaAla	VVV
rcsB	negative transcriptional regulator of flhDC (5)	p.His12Pro	GGG
		p.Glu34*	GGG
		p.His51fs	VVV
		p.Leu101Ile	GGG
		p.Gly111Asp	VVV
		p.Pro117fs	GGG
		p.Ala121fs	SSS
		p.Gly126fs	SSS
		p.Glu155Lys	AAA
		p.Gly165Val	AAA, GGG
		p.Leu175Pro	GGG
		p.Ser188Pro	VVV
		p.Leu193Pro	GGG
		p.Asp198Tyr	AAA
		p.Ile199Thr	GGG
***rflM* (*ecnR*)**	negative transcriptional regulator of flhDC (6)	p.Asp8Glu	GGG
		p.Leu136Pro	VVV
		insertion	AAA
fliO	flagellum-specific chaperone that favors the assembly of the fT3SS secretion pore (2)	p.Gln10Arg	SSS
		p.Trp38Arg	SSS
		p.Gly64Asp	SSS
lon	protease involved in FliP turnover	p.Asn578Lys	GGG
secA	ATPase of the Sec secretion pathway that is used by several fT3SS membrane proteins (including FliP) to assemble in the inner membrane	p.His262Pro	SSS

Table S3. List of strains and plasmids used in this study (without random mutants and suppressors)

Strain	Genotype	Source
TH437	*Salmonella enterica* serovar Typhimurium LT2	Kelly T. Hughes
TH10549	Δ*fliP6709* (Δaa6-241)	Kelly T. Hughes
TH10551	Δ*fliR6711* (Δaa6-260)	Kelly T. Hughes
TH12731	Δ*flgBC6557 flgE6569::bla* Δ*fliP7457*	Kelly T. Hughes
EM2225	*fliP22354*::G157-3×FLAG	(2)
EM3601–19<br>EM3621–39<br>EM3641–59	*fliP*::G157-3×FLAG M-loop mutants:<br>EM3601 (M209R), EM3602 (M209H), EM3603 (M209K), EM3604 (M209D), EM3605 (M209E),<br>EM3606 (M209S), EM3607 (M209T), EM3608 (M209N), EM3609 (M209Q), EM3610 (M209C),<br>EM3611 (M209G), EM3612 (M209P), EM3613 (M209A), EM3614 (M209V), EM3615 (M209I),<br>EM3616 (M209L), EM3617 (M209F), EM3618 (M209Y), EM3619 (M209W),<br>EM3621 (M210R), EM3622 (M210H), EM3623 (M210K), EM3624 (M210D), EM3625 (M210E),<br>EM3626 (M210S), EM3627 (M210T), EM3628 (M210N), EM3629 (M210Q), EM3630 (M210C),<br>EM3631 (M210G), EM3632 (M210P), EM3633 (M210A), EM3634 (M210V), EM3635 (M210I),<br>EM3636 (M210L), EM3637 (M210F), EM3638 (M210Y), EM3639 (M210W),<br>EM3641 (M211R), EM3642 (M211H), EM3643 (M211K), EM3644 (M211D), EM3645 (M211E),<br>EM3646 (M211S), EM3647 (M211T), EM3648 (M211N), EM3649 (M211Q), EM3650 (M211C),<br>EM3651 (M211G), EM3652 (M211P), EM3653 (M211A), EM3654 (M211V), EM3655 (M211I),<br>EM3656 (M211L), EM3657 (M211F), EM3658 (M211Y), EM3659 (M211W)	This study
EM5696–709<br>EM5718<br>EM5723–54<br>EM5788–96<br>EM6122–3	Δ*flgBC6557 flgE6569::bla fliP*::G157-3×FLAG M-loop mutants:<br>EM5696 (M209R), EM5697 (M209H), EM6122 (M209K), EM5723 (M209D), EM6123 (M209E),<br>EM5698 (M209S), EM5724 (M209T), EM5725 (M209N), EM5726 (M209Q), EM5699 (M209C),<br>EM5727 (M209G), EM5789 (M209P), EM5790 (M209A), EM5700 (M209V), EM5728 (M209I),<br>EM5729 (M209L), EM5791 (M209F), EM5730 (M209Y), EM5701 (M209W),<br>EM5731 (M210R), EM5702 (M210H), EM5703 (M210K), EM5792 (M210D), EM5732 (M210E),<br>EM5733 (M210S), EM5734 (M210T), EM5704 (M210N), EM5735 (M210Q), EM5736 (M210C),<br>EM5737 (M210G), EM5738 (M210P), EM5739 (M210A), EM5795 (M210V), EM5740 (M210I),<br>EM5705 (M210L), EM5796 (M210F), EM5741 (M210Y), EM5949 (M210W),<br>EM5706 (M211R), EM5742 (M211H), EM5707 (M211K), EM5743 (M211D), EM5744 (M211E),<br>EM5708 (M211S), EM5745 (M211T), EM5746 (M211N), EM5709 (M211Q), EM5747 (M211C),<br>EM5748 (M211G), EM5749 (M211P), EM5750 (M211A), EM5718 (M211V), EM5788 (M211I),<br>EM5751 (M211L), EM5752 (M211F), EM5753 (M211Y), EM5754 (M211W)	This study
EM3987–9<br>TR81–3	*fliP*::G157-3×FLAG M-loop M/A/L variation:<br>EM3987 (MLL), EM3988 (LML), EM3989 (LLM), TR81 (MAA), TR82 (AMA), TR83 (AAM)	This study
EM3035<br>EM3748–50	*fliP*::G157-3×FLAG triple M-loop mutants:<br>EM3035 (AAA), EM3748 (FFF), EM3749 (LLL), EM3750 (VVV),<br>EM7812 (GGG), EM7813 (SSS), EM7814 (III), EM7948 (CCC)	This study
EM3751	*fliP*::G157-3×FLAG V212M	This study
EM4044–7<br>EM6247	Δ*hin-5718*::FRT *fljB5001*::MudA *fliP*::G157-3×FLAG triple M-loop mutants:<br>EM4044 (GGG), EM4045 (SSS), EM4046 (III), EM4047 (CCC),<br>EM6247 (LLL)	This study
EM3132<br>EM6963–9	Δ*flgBC6557 flgE6569::bla fliP*::G157-3×FLAG M-loop mutants:<br>EM3132 (AAA), EM6963 (VVV), EM6964 (III), EM6965 (LLL),<br>EM6966 (FFF), EM6967 (GGG), EM6968 (CCC), EM6969 (SSS)	This study
EM9374–9<br>EM9395–6<br>EM9413–8<br>EM9423–4	*flgE6569::bla* Δ*flgBC6557 fliP* M-loop mutants ± flagellar deletions:<br>EM9375 (MMM + Δ*flhA7453*), EM9374 (AAA + Δ*flhA7453*),<br>EM9423 (MMM + Δ*flhA23039*), EM9424 (AAA + Δ*flhA23039*),<br>EM9377 (MMM + Δ*flhB7456*), EM9376 (AAA + Δ*flhB7456*),<br>EM9379 (MMM + Δ*flhB23038*), EM9378 (AAA + Δ*flhB23038*),<br>EM9396 (MMM + ΔflhBAE7670), EM9395 (AAA + ΔflhBAE7670),<br>EM9413 (MMM + Δ*fliF7387*), EM9414 (AAA + Δ*fliF7387*),<br>EM9415 (MMM + Δ*fliI7395*), EM9416 (AAA + Δ*fliI7395*),<br>EM9417 (MMM + ΔfliHIJ7398), EM9418 (AAA + ΔfliHIJ7398),<br>EM11333 (MAA + *fliR* Δ109-120::SGS), EM11592 (EGR), EM11707 (Δ109-120::SGS)	This study
EM11641—54<br>EM11675<br>EM11785–6<br>EM11793–4	*flgE6569::bla* Δ*flgBC6557 flhA23108*::SAGASA-mNeonGreen + *fliP* M-loop mutants:<br>EM11641 (ΔfliP), EM11642 (AAA), EM11643 (AMM), EM11644 (MAM), EM11645 (MMA),<br>EM11646 (WMM), EM11647 (MWM), EM11648 (MMW), EM11649 (RMM), EM11650 (MEM),<br>EM11651 (MMR), EM11652 (MMD), EM11653 (MKM), EM11654 (MAA), EM11675 (MMM),<br>EM11685 (MAA + *flhA* D158N), EM11686 (MMM + *flhA* D158N), EM11693 (EGR)	This study

Table continues on the next page

Strain	Genotype	Source
TR46	Δ*hin-5718*::FRT *fljB5001*::MudA *fliP* G157 3×FLAG M insertion in M-loop (4M)	This study
TR88–93<br>TR121–2	*fliQ* mutants:<br>TR88 (Q39A), TR89 (E46A), TR90 (K54A), TR91 (E46D),<br>TR92 (M47R), TR93 (M47Q), TR121 (M47A), TR122 (M47C)	This study
TR123–5<br>TR222-4<br>TR228<br>TR299–300	*fliP* G157::3×FLAG XXX M-loop mutants + *fliQ* E46D combinations:<br>TR123 (AAA + E46D), TR124 (CCC + E46D), TR125 (GGG + E46D),<br>TR222 (FFF + E46D), TR223 (SSS + E46D), TR228 (VVV + E46D),<br>TR299 (III + E46D), TR300 (LLL + E46D)	This study
TR143–8	*fliR* plug mutants (substitutions of residues 109–120):<br>TR143 (SAATAADGGSSA), TR144 (SAATAASGGSSA = flexible plug),<br>TR145 (SGS = Δplug), TR146 (SGSGS), TR147 (SGSGSGS), TR148 (D115A)	This study
TR183	*fliP*::G157-3×FLAG (ΔM)	This study
TR186	*fliP*::G157-3×FLAG Δ*fliR6711* (Δaa6-260)	This study
TR189	*fliP*::G157-3×FLAG *fliR* flexible plug (Δ109–120::SAATAASGGSSA)	This study
TR192	*fliP*::G157-3×FLAG *fliR* Δplug (Δ109–120::SGS)	This study
TR197–8<br>EM2716	Δ*flgBC6557 flgE6569::bla* Δ*rflP252 fliP*::G157- 3×FLAG M-loop variants:<br>EM2716(MMM), TR197 (AAA), TR198 (FFF)	This study
TR266–73	*fliP* G157::3×Flag MXX M-loop mutants:<br>TR266 (MVV), TR267 (MYY), TR268 (MTT), TR269 (MFF), TR270 (MII), TR271 (MWW),<br>TR272 (MGG), TR273 (MSS)	This study
TR291	*fliP*::G157-3×FLAG (MMM209MAA) *fliQ* (M47A)	This study
TR297	*flgE6569::bla* Δ*flgBC6557 fliR* Δplug (Δ109–120::SGS)	This study
TR317–20	*fliP*::G157- 3×FLAG M-loop mutants combinations with FliR plug mutants:<br>TR317 (MAA + Δplug), TR318 (MFF + Δplug), TR319 (MSS + Δplug), TR320 (MWW +<br>Δplug)	This study
TR321–3<br>TR328–30	*flgE6569::bla* Δ*flgBC6557 fliP* G157::3×Flag M-loop mutants:<br>TR321 (MAA), TR322 (ΔM), TR323 (ΔMM), TR328 (MWW), TR329 (MGG), TR330 (MSS),<br>TR330 (MFF)	This study
	plasmids used for *fliQ* tryptophane scanning derive from the pMS11 (7) with the following mutations: L24W, L25W, L24W, L26W, V27W, A28W, L29W, I60W, A61W, I62W, I63W, V64W, A65W, G66W	This study

Table S4. List of primers used in this study. Capital letters indicate nucleotides that were mutated, or homology to the Kan-SceI cassette. The regions coding for the *fliP* M-loop and the *fliR* R-plug are shown in bold.

Gene	Mutation	Primer name	Primer sequence
fliP	fliP::Kan-SceI	FliP_M209_Kan_FW	tatcgacctggtgatcgccagcgtattgatggcgttggggAGGGTTTTCCCAGTCACGAC
	fliP::Kan-SceI	FliP_M211_Kan_RV	gcatcagcttaaacggtagggcgattgtcgctggcggcactGCTTCCGGCTCGTATGTTG
	M209X (NNS)	FliP_M209_NNS_FW	tatcgacctggtgatcgccagcgtattgatggcgttgggg**NNSatgatg**gtgccgccagc
	M210X (NNC)	FliP_M210_NNC_FW	cgacctggtgatcgccagcgtattgatggcgttgggg**atgNNCatg**gtgccgccagcgac
	M211X (NNS)	FliP_M211_NNS_FW	ctggtgatcgccagcgtattgatggcgttgggg**atgatgNNS**gtgccgccagcgacaatc
	M209K	FliP_M209K_FW	gattatcgacctggtgatcgccagcgtattgatggcgttgggg**AAgatgatg**gtgccgccagcgac
	M209E	FliP_M209E_FW	gattatcgacctggtgatcgccagcgtattgatggcgttgggg**GAgatgatg**gtgccgccagcgac
	M209R	FliP_M209R_FW	gattatcgacctggtgatcgccagcgtattgatggcgttgggg**CGTatgatg**gtgccgccagcgac
	M209T	FliP_M209T_FW	gattatcgacctggtgatcgccagcgtattgatggcgttgggg**aCgatgatg**gtgccgccagcgac
	M210K	FliP_M210K_FW	gtattgatggcgttgggg**atgaAgatg**gtgccgccagcgacaatc
	M210Q	FliP_M210Q_FW	gattatcgacctggtgatcgccagcgtattgatggcgttgggg**atgCAgatg**gtgccgccagcgacaatc
	M210W	FliP_M210W_FW	gattatcgacctggtgatcgccagcgtattgatggcgttgggg**atgTGgatg**gtgccgccagcgacaatc
	M210S	FliP_M210S_FW	gattatcgacctggtgatcgccagcgtattgatggcgttgggg**atgaGTatg**gtgccgccagcgacaatc
	M210T	FliP_M210T_FW	gattatcgacctggtgatcgccagcgtattgatggcgttgggg**atgaCgatg**gtgccgccagcgacaatc
	M210F	FliP_M210F_FW	gattatcgacctggtgatcgccagcgtattgatggcgttgggg**atgTtCatg**gtgccgccagcgacaatc
	MMM209MAA	FliP_MMM209MAA_FW	gattatcgacctggtgatcgccagcgtattgatggcgttgggg**atgGCgGCg**gtgccgccagcgacaatc
	MMM209AMA	FliP_MMM209AMA_FW	gattatcgacctggtgatcgccagcgtattgatggcgttgggg**GCgatgGCg**gtgccgccagcgacaatc
	MMM209AAM	FliP_MMM209AAM_FW	gattatcgacctggtgatcgccagcgtattgatggcgttgggg**GCgGCgatg**gtgccgccagcgacaatc
	MMM209MLL	FliP_MMM209MLL_FW	cgacctggtgatcgccagcgtattgatggcgttgggg**atgCTGCTG**gtgccgccagcgac
	MMM209LML	FliP_MMM209LML_FW	cgacctggtgatcgccagcgtattgatggcgttgggg**CTGatgCTG**gtgccgccagcgac
	MMM209LLM	FliP_MMM209LLM_FW	cgacctggtgatcgccagcgtattgatggcgttgggg**CTGCTGatg**gtgccgccagcgac
	MMM209AAA	FliP_MMM209AAA_FW	gtattgatggcgttgggg**GCAGCCGCG**gtgccgccagcgacaatcgccctacc
	MMM209VVV	FliP_MMM209VVV_FW	cgacctggtgatcgccagcgtattgatggcgttgggg**GTGGTGGTG**gtgccgccagcgac
	MMM209LLL	FliP_MMM209LLL_FW	cgacctggtgatcgccagcgtattgatggcgttgggg**CTGCTGCTG**gtgccgccagcgac
	MMM209FFF	FliP_MMM209FFF_FW	cgacctggtgatcgccagcgtattgatggcgttgggg**TTCTTCTTC**gtgccgccagcgac
	MMM209GGG	FliP_MMM209GGG_FW	cgacctggtgatcgccagcgtattgatggcgttgggg**GGAGGAGGA**gtgccgccagcgac
	MMM209III	FliP_MMM209III_FW	cgacctggtgatcgccagcgtattgatggcgttgggg**ATCATCATC**gtgccgccagcgac
	MMM209CCC	FliP_MMM209CCC_FW	cgacctggtgatcgccagcgtattgatggcgttgggg**TGTTGTTGT**gtgccgccagcgac
	MMM209SSS	FliP_MMM209SSS_FW	cgacctggtgatcgccagcgtattgatggcgttgggg**AGCAGCAGC**gtgccgccagcgac
	V212M	FliP_V212M_FW	ctggtgatcgccagcgtattgatggcgttgggg**atgatgatg**Atgccgccagcgacaatc
	4M (insertion)	FliP_4M_FW	cccttttttgattatcgacctggtgatcgccagcgtattgatggcgttgggg**ATGATGATGATG**gtgccgccagcgacaatcgccctaccgtttaagctgatg
	MMM209MSS	FliP_MMM209MSS_FW	gattatcgacctggtgatcgccagcgtattgatggcgttgggg**atgaCgaCg**gtgccgccagcgacaatc
	MMM209MFF	FliP_MMM209MFF_FW	gattatcgacctggtgatcgccagcgtattgatggcgttgggg**atgTtCTtC**gtgccgccagcgacaatc
	MMM209MGG	FliP_MMM209MGG_FW	gattatcgacctggtgatcgccagcgtattgatggcgttgggg**atgGGgGGg**gtgccgccagcgacaatc
	MMM209MII	FliP_MMM209MII_FW	gattatcgacctggtgatcgccagcgtattgatggcgttgggg**atgatCatC**gtgccgccagcgacaatc
	MMM209MVV	FliP_MMM209MVV_FW	gattatcgacctggtgatcgccagcgtattgatggcgttgggg**atgGtgGtg**gtgccgccagcgacaat
	MMM209MYY	FliP_MMM209MYY_FW	gattatcgacctggtgatcgccagcgtattgatggcgttgggg**atgTACTAC**gtgccgccagcgacaat
	MMM209MWW	FliP_MMM209MWW_FW	gattatcgacctggtgatcgccagcgtattgatggcgttgggg**atgTGgTGg**gtgccgccagcgacaat
	random M-loop	FliP_MMM209(NNS)3_FW	cgacctggtgatcgccagcgtattgatggcgttgggg**NNSNNSNNS**gtgccgccagcgac
fliQ	fliQ::Kan-SceI	FliQ_L38_Kan_FW	actcgtcgcgctgattaccggcctcattatcagcatcttgAGGGTTTTCCCAGTCACGAC
	fliQ::Kan-SceI	FliQ_N45_Kan_FW	cctcattatcagcatcttgcaggccgcgactcagattaatAGGGTTTTCCCAGTCACGAC
	fliQ::Kan-SceI	FliQ_I55_Kan_RV	acgggccggcaacgataattgcgatgaataccgcgacgatTGCTTCCGGCTCGTATGTTG
	Q39A	FliQ_Q39A_FW	actcgtcgcgctgattaccggcctcattatcagcatcttgGCggccgcgactcagattaa
	E46A	FliQ_E46A_FW	cctcattatcagcatcttgcaggccgcgactcagattaatgCaatgacgctgtcgtttat
	K54A	FliQ_K54A_RV	acgggccggcaacgataattgcgatgaataccgcgacgattGCagggataaacgacagcg
	E46D	FliQ_E46D_FW	ctcattatcagcatcttgcaggccgcgactcagattaatgaTatgacgctgtcgtttatc
	M47R	FliQ_M47R_FW	ctcattatcagcatcttgcaggccgcgactcagattaatgaaCGgacgctgtcgtttatc
	M47Q	FliQ_M47Q_FW	ctcattatcagcatcttgcaggccgcgactcagattaatgaaCAgacgctgtcgtttatc
	M47A	FliQ_M47A_FW	ctcattatcagcatcttgcaggccgcgactcagattaatgaaGCgacgctgtcgtttatc
	M47C	FliQ_M47C_FW	ctcattatcagcatcttgcaggccgcgactcagattaatgaaTGCacgctgtcgtttatc
fliR	fliR::Kan-SceI	FliR_109_Kan_FW	gcgtaccgcaggtgagtttatcggtctgcaaatggggcttAGGGTTTTCCCAGTCACGAC
	fliR::Kan-SceI	FliR_120_Kan_RV	tggcgagcatatccataatacgcgccagaaccggcatattTGCTTCCGGCTCGTATGTTG
	flexible plug	FliR_SAATAASGGSSA_FW	gcgtaccgcaggtgagtttatcggtctgcaaatggggctt**tccGCGgccaccGCcgCcAGcGGCggcagcTCcGCg**aatatgccggttctggcg
	flexible plug 2	FliR_SAATAADGGSSA_FW	gcgtaccgcaggtgagtttatcggtctgcaaatggggctt**tccGCGgccaccGCcgCcgacGGCggcagcTCcGCg**aatatgccggttctggcg
	plug deletion	FliR_plug_SGS_FW	gcgtaccgcaggtgagtttatcggtctgcaaatggggctt**tccGGtAGc**aatatgccggttctggcg
	plug deletion 2	FliR_plug_SGSGS_FW	gcgtaccgcaggtgagtttatcggtctgcaaatggggctt**tccGGtAGcGGctCc**aatatgccggttctggcg
	plug deletion 3	FliR_plug_SGSGSGS_FW	gcgtaccgcaggtgagtttatcggtctgcaaatggggctt**tccGGtAGcGGctCcgGcAGc**aatatgccggttctggcg
	D115A	FliR_D115A_FW	gcgtaccgcaggtgagtttatcggtctgcaaatggggctt**tcctttgccaccttcgtcgCcccgggcagccacctg**aatatg

Supplementary references

1. Elizabeth Ward, Thibaud T. Renault, Eun A. Kim, Marc Erhardt, Kelly T. Hughes, and David F. Blair. Type-III Secretion Pore Formed by Flagellar Protein FliP. *Molecular Microbiology*, October 2017. ISSN 1365-2958. doi:10.1111/mmi.13870.
2. Florian D. Fabiani, Thibaud T. Renault, Britta Peters, Tobias Dietsche, Eric J. C. Gálvez, Alina Guse, Karen Freier, Emmanuelle Charpentier, Till Strowig, Mirita Franz-Wachtel, Boris Macek, Samuel Wagner, Michael Hensel, and Marc Erhardt. A flagellum-specific chaperone facilitates assembly of the core type III export apparatus of the bacterial flagellum. *PLoS biology*, 15(8):e2002267, August 2017. ISSN 1545-7885. doi:10.1371/journal.pbio.2002267.
3. William S. J. Valdar. Scoring residue conservation. *Proteins: Structure, Function, and Bioinformatics*, 48(2):227–241, 2002. ISSN 1097-0134. doi:10.1002/prot.10146.
4. D. Lehnen, C. Blumer, T. Polen, B. Wackwitz, V. F. Wendisch, and G. Unden. LrhA as a new transcriptional key regulator of flagella, motility and chemotaxis genes in *Escherichia coli*. *Molecular Microbiology*, 45(2):521–532, July 2002. ISSN 0950-382X. doi:10.1046/j.1365-2958.2002.03032.x.
5. Anne Francez-Charlot, Bruno Laugel, Alice Van Gemert, Nelly Dubarry, Florent Wiorowski, Marie-Pierre Castanié-Cornet, Claude Gutierrez, and Kaymeuang Cam. RcsCDB His-Asp phosphorelay system negatively regulates the flhDC operon in *Escherichia coli*. *Molecular Microbiology*, 49(3):823–832, August 2003. ISSN 0950-382X. doi:10.1046/j.1365-2958.2003.03601.x.
6. Christopher E. Wozniak, Changhan Lee, and Kelly T. Hughes. T-POP Array Identifies EcnR and PefI-SrgD as Novel Regulators of Flagellar Gene Expression. *Journal of Bacteriology*, 191(5): 1498–1508, March 2009. ISSN 0021-9193, 1098-5530. doi:10.1128/JB.01177-08.
7. Marc Erhardt, Paige Wheatley, Eun A. Kim, Takanori Hirano, Yang Zhang, Mayukh K. Sarkar, Kelly T. Hughes, and David F. Blair. Mechanism of type-III protein secretion: Regulation of FlhA conformation by a functionally critical charged-residue cluster. *Molecular Microbiology*, 104(2):234–249, April 2017. ISSN 1365-2958. doi:10.1111/mmi.13623.
