# Supplementary material for: Control of membrane barrier during bacterial type-III protein secretion
Source: Nat Commun. 2021 Jun 28;12:3999. doi: 10.1038/s41467-021-24226-1 (PMC8239009; doi:10.1038/s41467-021-24226-1)
Supplement: Supplementary file 3 — Reporting Summary [file 41467_2021_24226_MOESM3_ESM.pdf]

nature research

Corresponding author(s): Thibaud Renault and Marc Erhardt

Last updated by author(s): May 27, 2021

Reporting Summary

Nature Research wishes to improve the reproducibility of the work that we publish. This form provides structure for consistency and transparency in reporting. For further information on Nature Research policies, see our Editorial Policies and the Editorial Policy Checklist.

Statistics

For all statistical analyses, confirm that the following items are present in the figure legend, table legend, main text, or Methods section.

n/a	Confirmed	
☐	☒	The exact sample size (*n*) for each experimental group/condition, given as a discrete number and unit of measurement
☐	☒	A statement on whether measurements were taken from distinct samples or whether the same sample was measured repeatedly
☐	☒	The statistical test(s) used AND whether they are one- or two-sided<br>*Only common tests should be described solely by name; describe more complex techniques in the Methods section.*
☒	☐	A description of all covariates tested
☐	☒	A description of any assumptions or corrections, such as tests of normality and adjustment for multiple comparisons
☐	☒	A full description of the statistical parameters including central tendency (e.g. means) or other basic estimates (e.g. regression coefficient) AND variation (e.g. standard deviation) or associated estimates of uncertainty (e.g. confidence intervals)
☐	☒	For null hypothesis testing, the test statistic (e.g. *F*, *t*, *r*) with confidence intervals, effect sizes, degrees of freedom and *P* value noted<br>*Give P values as exact values whenever suitable.*
☒	☐	For Bayesian analysis, information on the choice of priors and Markov chain Monte Carlo settings
☒	☐	For hierarchical and complex designs, identification of the appropriate level for tests and full reporting of outcomes
☐	☒	Estimates of effect sizes (e.g. Cohen's *d*, Pearson's *r*), indicating how they were calculated

Our web collection on statistics for biologists contains articles on many of the points above.

Software and code

Policy information about availability of computer code

Data collection	Plate reader: Biotek Gen5 software<br>Microscopy: Zeiss Zen 2.6 pro software, Nikon NIS-Elements AR 5.30.02 (64-bit)<br>Scanning of motility plates: python 3.5, pyinsane 1.4.0
Data analysis	Data was processed using pymol 2.5, ImageJ 1.52i, MicrobeJ, and analyzed using python 3.8 (numpy 560 1.19.2, pandas 1.1, matplotlib 3.3, seaborn 0.11, scipy 1.5) Protein logos were generated using weblogo 561 3.7.5. The phylogenic tree of the M-loop mutants was graphed using iTOL 5.7.

For manuscripts utilizing custom algorithms or software that are central to the research but not yet described in published literature, software must be made available to editors and reviewers. We strongly encourage code deposition in a community repository (e.g. GitHub). See the Nature Research guidelines for submitting code & software for further information.

Data

Policy information about availability of data

All manuscripts must include a data availability statement. This statement should provide the following information, where applicable:

- Accession codes, unique identifiers, or web links for publicly available datasets
- A list of figures that have associated raw data
- A description of any restrictions on data availability

The source data and the full scans of the blots are provided as a Source Data file. Biological materials and other data are available upon reasonable request to the corresponding authors. We have added this information in the "Data availability" statement, which reads:
"The coordinates of the FliPQR complex in its closed state are available at the Protein Data Bank under the accession number 6f2d. The source data of all relevant

figures and the full scans of the blots in Figures 5c and S2a are available online as a Source Data file. Biological materials and other data underlying this article are available upon reasonable request to the corresponding authors."

Field-specific reporting

Please select the one below that is the best fit for your research. If you are not sure, read the appropriate sections before making your selection.

☒ Life sciences ☐ Behavioural & social sciences ☐ Ecological, evolutionary & environmental sciences

For a reference copy of the document with all sections, see nature.com/documents/nr-reporting-summary-flat.pdf

Life sciences study design

All studies must disclose on these points even when the disclosure is negative.

Sample size	No a priori sample sizes were calculated. Sample sizes were chosen according to our experience in similar experimental setups e.g. Santiveri, M. et al. (2020) Cell 183: 244-257.e16.; Ward et. al. (2018) Mol Microbiol 107: 94-103.; Spöring, I. et al. (2018) PLoS Biol 16: e2006989.; Renault, T.T. et al. (2017) Elife 6: e23136.; Fabiani, F.D. et al. (2017) PLoS Biol 15: e2002267. The exact sample size (n) are provided in the figure legends.
Data exclusions	No data was excluded from the analysis.
Replication	All reported experiments have been repeated at least 3 times with independent samples. All experimental results shown were reproducible. No exclusion criteria were pre-established.
Randomization	No experimental groups were formed/compared. Allocating experimental groups was not relevant for this study, as all bacterial cells of a particular strain are genetic clones.
Blinding	The researchers were not blinded to sample identity. Image and data analysis was automated wherever possible. Therfore, blinding was neither possible nor necessary for this study, as all bacterial cells of a particular strain are genetic clones and analyses were not sufficiently subjective to require researcher blinding.

Reporting for specific materials, systems and methods

We require information from authors about some types of materials, experimental systems and methods used in many studies. Here, indicate whether each material, system or method listed is relevant to your study. If you are not sure if a list item applies to your research, read the appropriate section before selecting a response.

Materials & experimental systems

n/a	Involved in the study	
☐	☒	Antibodies
☒	☐	Eukaryotic cell lines
☒	☐	Palaeontology and archaeology
☒	☐	Animals and other organisms
☒	☐	Human research participants
☒	☐	Clinical data
☒	☐	Dual use research of concern

Methods

n/a	Involved in the study	
☒	☐	ChIP-seq
☒	☐	Flow cytometry
☒	☐	MRI-based neuroimaging

Antibodies

Antibodies used	anti-FliC: Difco Salmonella H Antiserum I Difco 228241; anti-FljB: Difco Salmonella H Antiserum Single Factor 2 Difco 224741; anti-FLAG M2 affinity-purified Sigma F1804; anti-FlhAc: gift from Tohru Minamino (Minamino, T. et al. (2011) Nat Commun 2: 475.) secondary antibodies: Invitrogen anti-rabbit Alexa-Fluor488 (catalog number A-11094), Bio-Rad Immun-Star Goat anti-rabbit (GAR)-HRP conjugate (catalog number 170-5046), Bio-Rad Immun-Star Goat anti-mouse-HRP (catalog number 170-5047).
Validation	Primary antibodies were validated against Salmonella enterica serovar Typhimurium LT2 deletion mutants of the respective target proteins. Specificity of anti-FlhAc was validated using a flhA deletion mutant. Specificity of anti-FLAG was validated using wildtype Salmonella not expressing FLAG-epitope tagged proteins. The commercially available Difco Salmonella H Antisera are generally used in tube agglutination tests for the identification of Salmonella by flagellar (H) antigens and have been validated in our laboratory against phase-locked FliC-ON and FljB-ON strains, respectively.
