# Supplementary material for: Control of membrane barrier during bacterial type-III protein secretion
Source: Nat Commun. 2021 Jun 28;12:3999. doi: 10.1038/s41467-021-24226-1 (PMC8239009; doi:10.1038/s41467-021-24226-1)

FliP M209 mutants

TCE stain

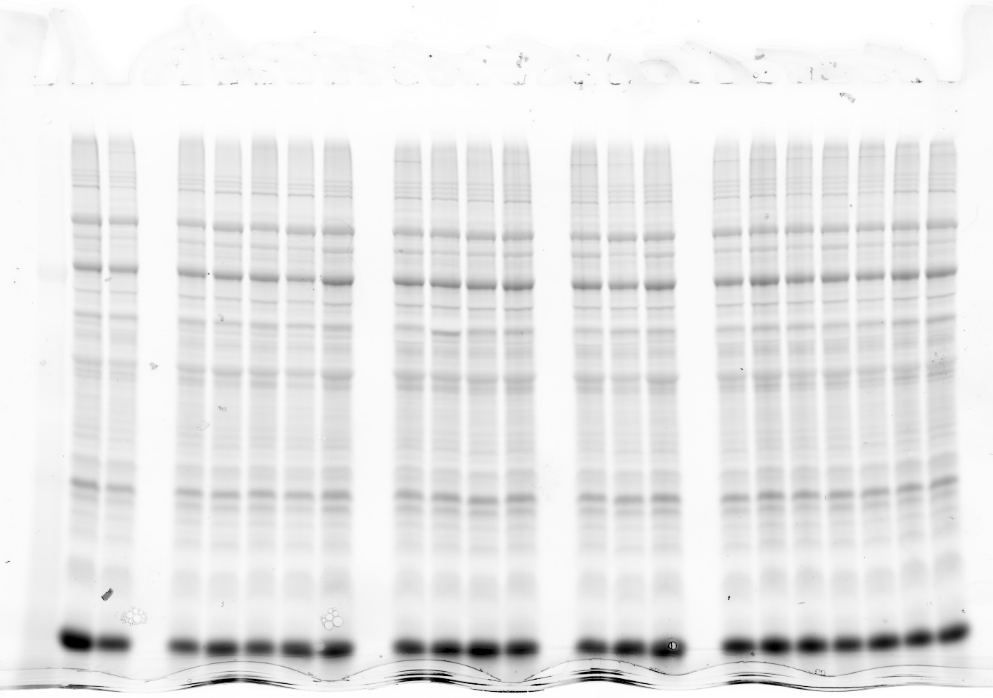

membrane / chemiluminescence overlay

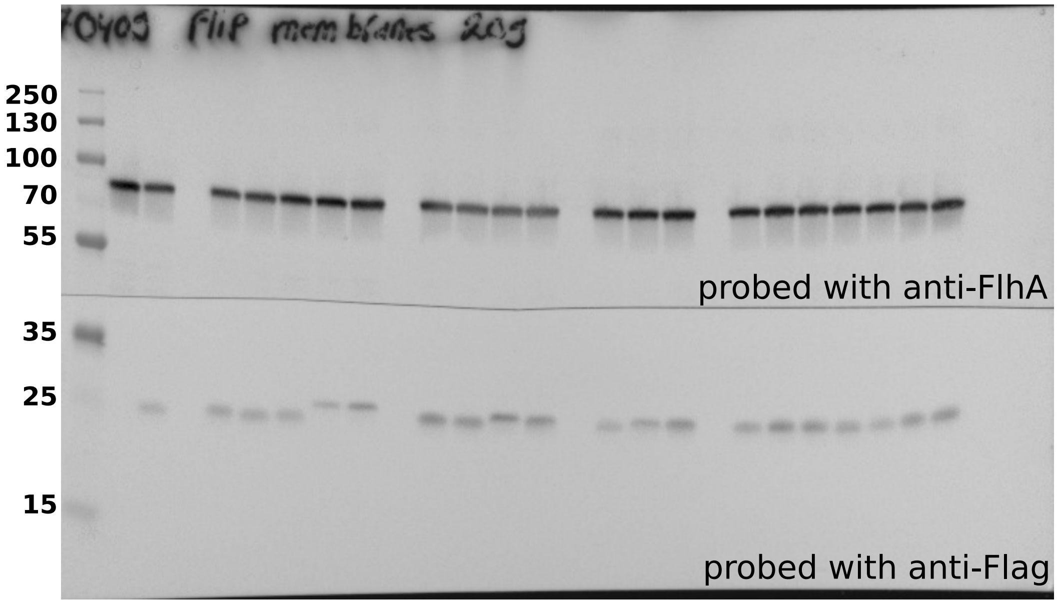

chemiluminescence

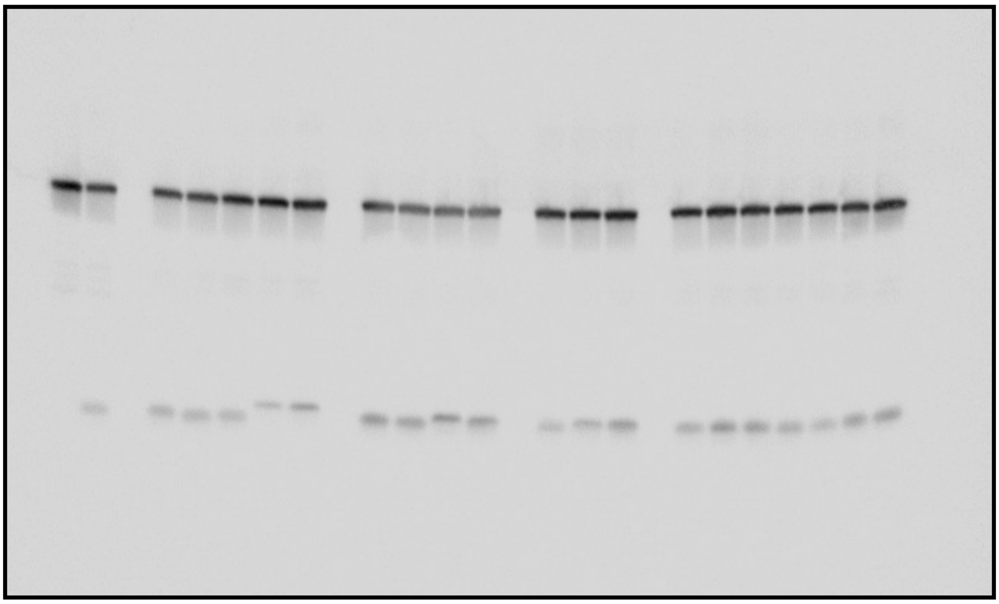

FliP M210 mutants

TCE stain

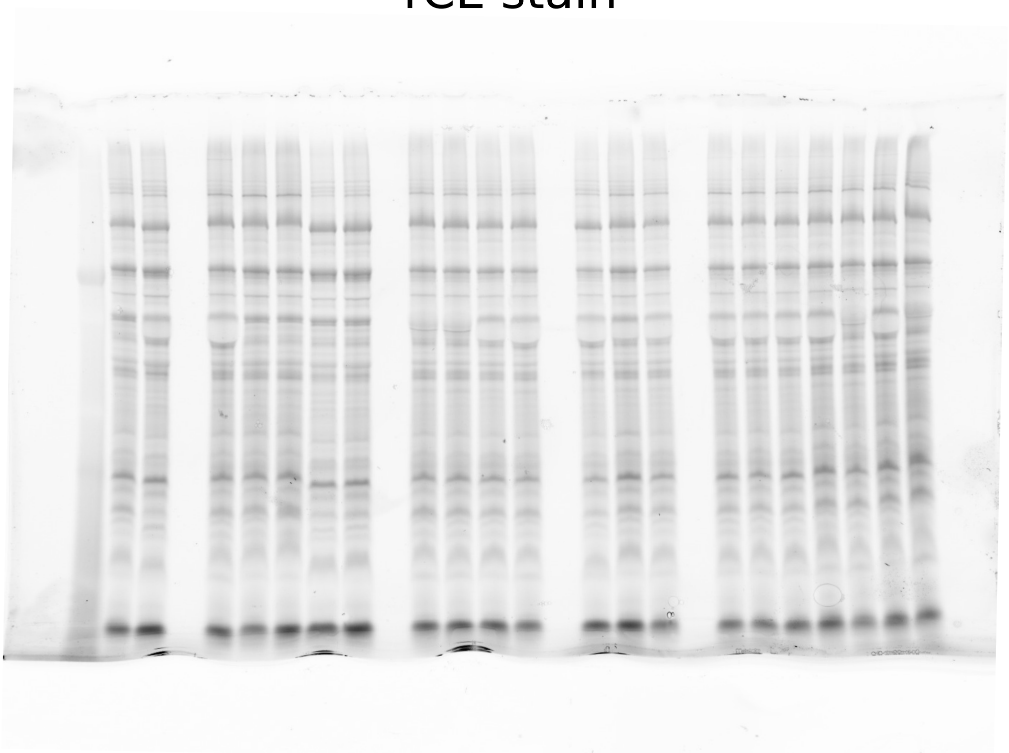

membrane / chemiluminescence overlay

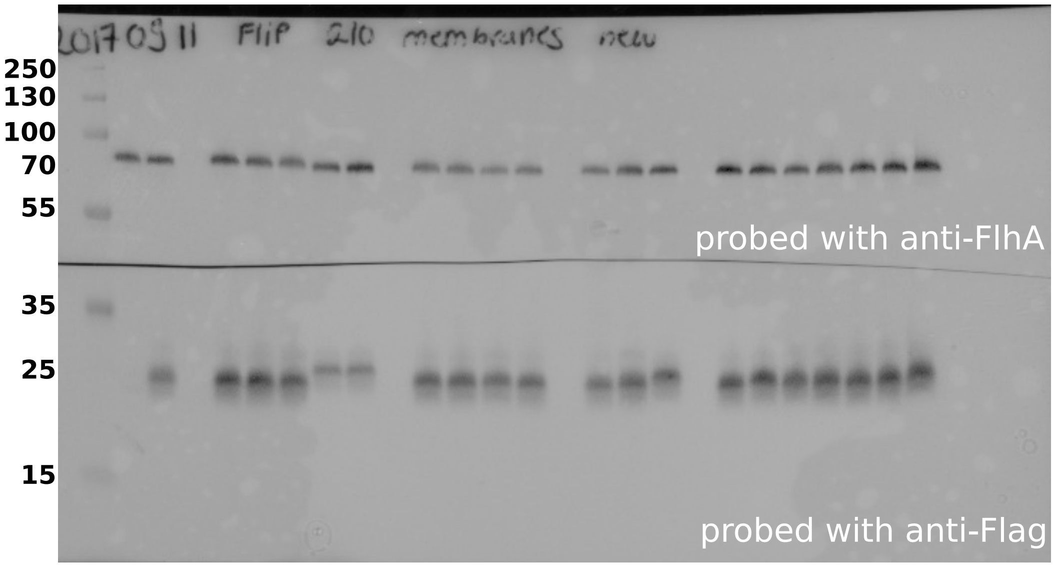

chemiluminescence

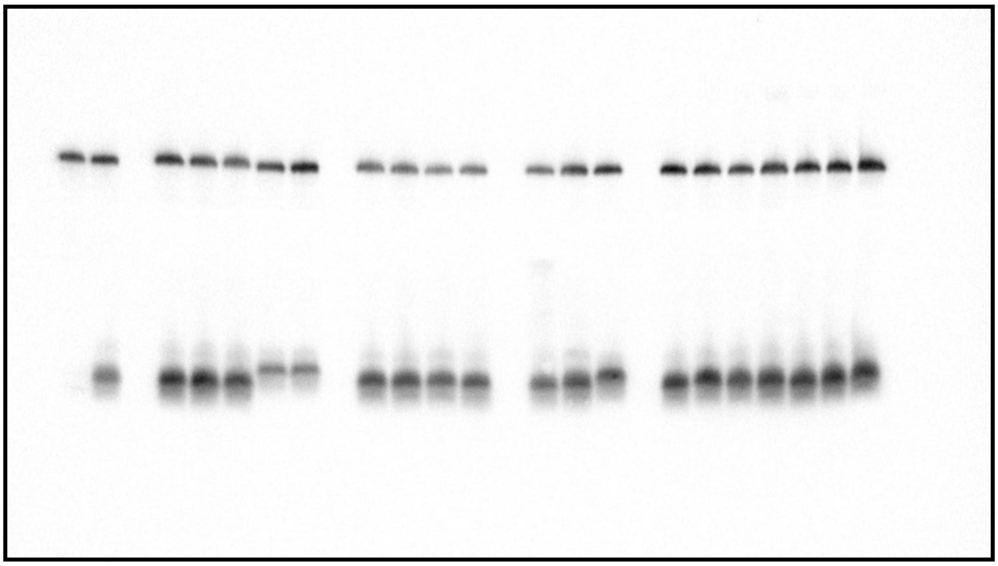

FliP M211 mutants

TCE stain

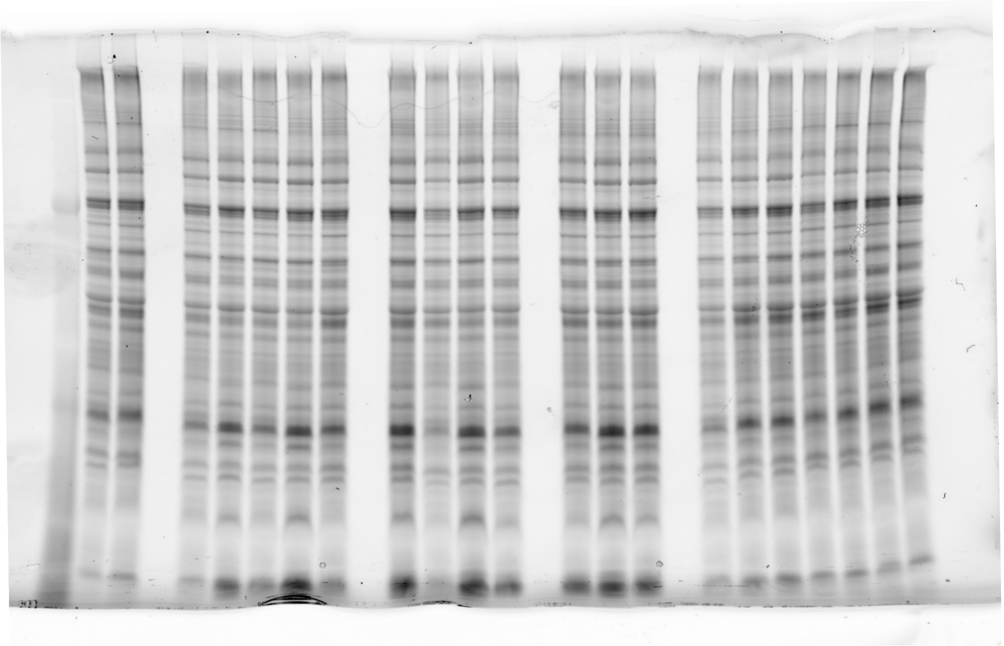

membrane / chemiluminescence overlay

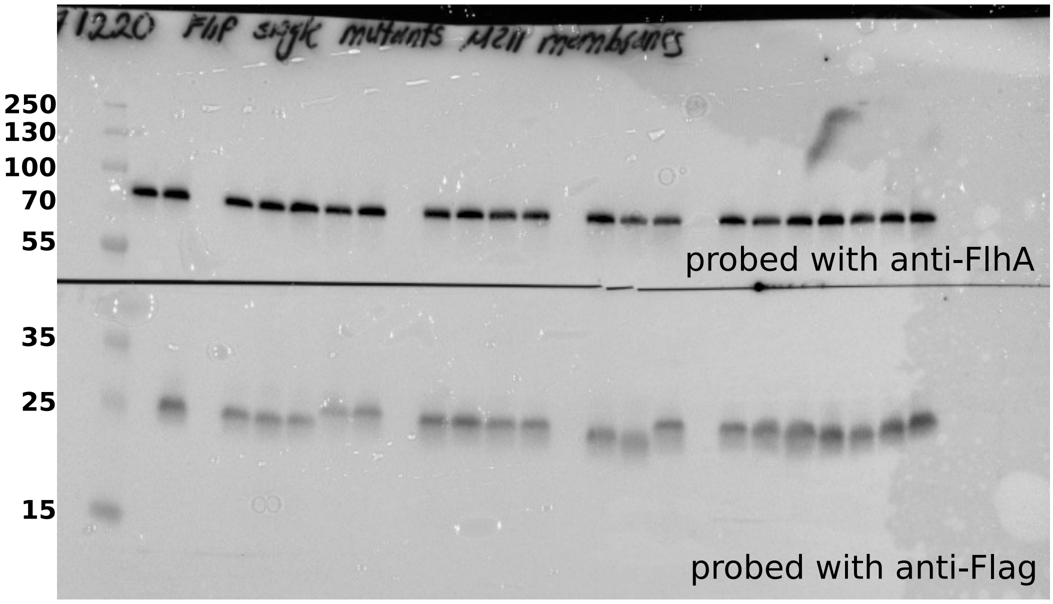

chemiluminescence

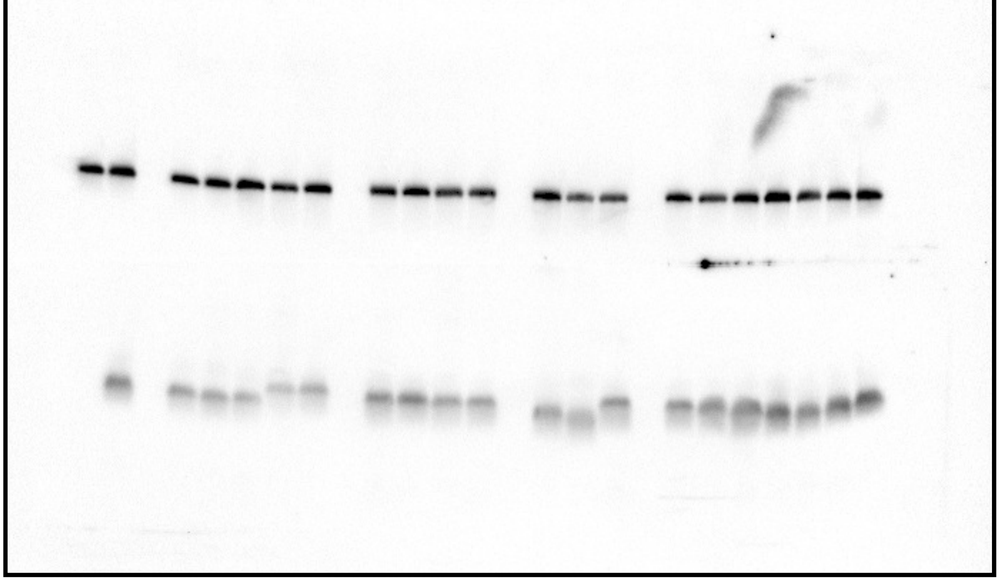

Supplement: Supplementary file 4 — Source Data [file 41467_2021_24226_MOESM4_ESM.zip › Source_Data/FigureS2a_full_blots.pdf]
